# Toxic Effects of Penetrating Cations

**DOI:** 10.3390/membranes13100841

**Published:** 2023-10-22

**Authors:** Svyatoslav Sokolov, Anna Zyrina, Sergey Akimov, Dmitry Knorre, Fedor Severin

**Affiliations:** 1 Department of Molecular Energetics of Microorganisms, Belozersky Institute of Physico-Chemical Biology, Lomonosov Moscow State University, 1–40 Leninskie Gory, 119991 Moscow, Russia; sokolss@belozersky.msu.ru (S.S.); knorre@belozersky.msu.ru (D.K.); 2 Chumakov Federal Scientific Center for Research and Development of Immune-and-Biological Products of Russian Academy of Sciences, Premises 8, Bldg. 1, Village of Institute of Poliomyelitis, Settlement “Moskovskiy”, 108819 Moscow, Russia; zyrina_an@chumakovs.su; 3 Frumkin Institute of Physical Chemistry and Electrochemistry, Russian Academy of Sciences, 31/4 LeninskiyProspekt, 119071 Moscow, Russia; akimov_sergey@mail.ru

**Keywords:** penetrating cations, mitochondria, membrane, uncoupler, phospholipid bilayer, anti-cancer

## Abstract

As mitochondria are negatively charged organelles, penetrating cations are used as parts of chimeric molecules to deliver specific compounds into mitochondria. In other words, they are used as electrophilic carriers for such chemical moieties as antioxidants, dyes, etc., to transfer them inside mitochondria. However, unmodified penetrating cations affect different aspects of cellular physiology as well. In this review, we have attempted to summarise the data about the side effects of commonly used natural (e.g., berberine) and artificial (e.g., tetraphenylphosphonium, rhodamine, methylene blue) penetrating cations on cellular physiology. For instance, it was shown that such types of molecules can (1) facilitate proton transport across membranes; (2) react with redox groups of the respiratory chain; (3) induce DNA damage; (4) interfere with pleiotropic drug resistance; (5) disturb membrane integrity; and (6) inhibit enzymes. Also, the products of the biodegradation of penetrating cations can be toxic. As penetrating cations accumulate in mitochondria, their toxicity is mostly due to mitochondrial damage. Mitochondria from certain types of cancer cells appear to be especially sensitive to penetrating cations. Here, we discuss the molecular mechanisms of the toxic effects and the anti-cancer activity of penetrating cations.

## 1. Introduction

There are two ways for lipophilic cations to cross a phospholipid bilayer. Single molecules of cations with small charged moieties (e.g., benzalkonium) cannot penetrate the bilayer because, in the aqueous phase, the moiety is attached to a number of water molecules via hydrogen bonds. Thus, such an aggregate cannot penetrate between the phospholipid molecules for sterical reasons. Additionally, the electrostatic Born energy of an ion in a dielectric medium (membrane) scales as the inverse ion radius [1]; for this reason, smaller ions have higher electrostatic energy inside the membrane as compared to larger ones, although for non-spherical molecules the membrane permeability strongly depends on the details of their molecular shape [2]. Still, such lipophilic molecules can cross the membrane via transient pores. If the effective cross-section of the polar parts of the ions is larger than the cross-section of their hydrophobic tail (that is usually the case), they locally induce a convex patch in the membrane monolayer, i.e., induce a positive spontaneous curvature [3,4]. Such curvature generally favours the formation of pores [5], and such cations can act as detergents. If the cation surface concentration increases, the curvature of the monolayer changes, leading to the formation of the pore [6]. Next, the molecules diffuse to the opposite monolayer, and the pore closes spontaneously. A similar effect was observed for the amphipathic peptide PGLa [7]. Upon incorporation into the outer monolayer of the membrane of giant unilamellar vesicles (GUV), this peptide makes the membrane locally convex. An increase in the peptide concentration leads to the formation of small transient pores, via which the peptide passes to the inner monolayer of the GUV membrane [7].

**Figure 1:**
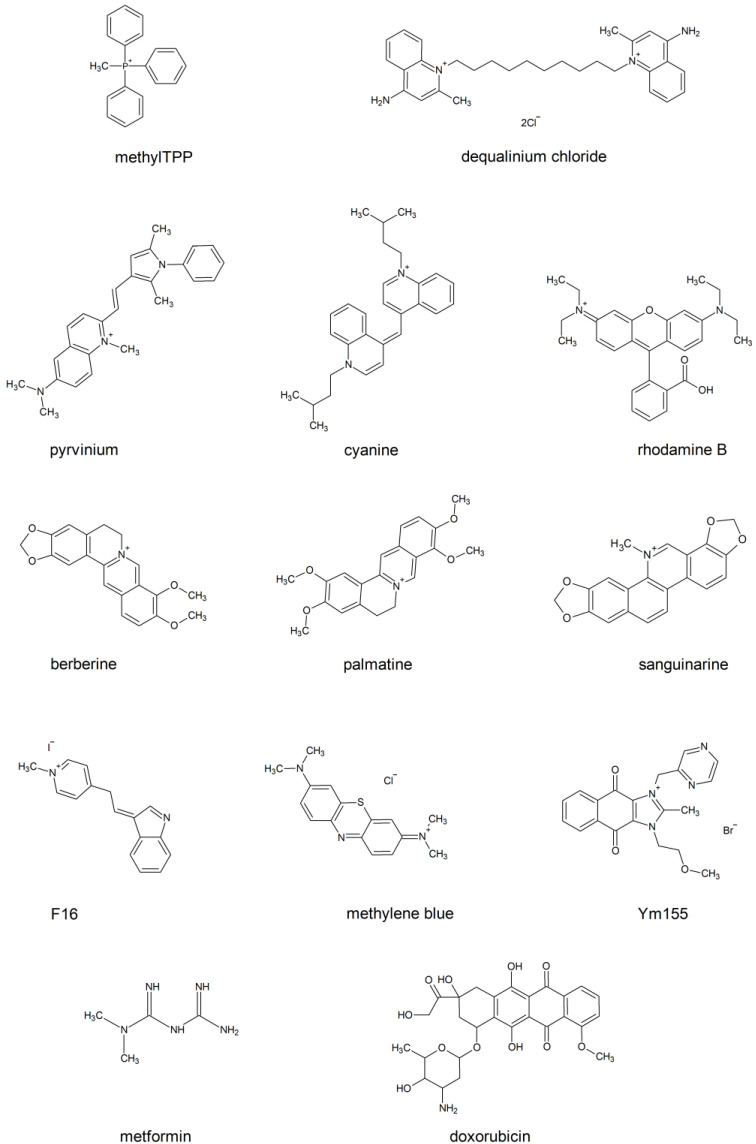
Сhemical structures of lipocations.

An alternative way is to cross without disrupting the integrity of the lipid bilayer. The lipocations, which lack the voluminous layer of hydrogen bond-attached water molecules, are capable of this. To avoid the formation of the halo of water molecules, the charge has to be either masked by hydrophobic groups, or delocalized, or both (e.g., triphenylphosphonium (TPP) and rhodamine, respectively, Figure 1). The latter group of lipocations is likely to be less harmful for the biological membranes than the former one. Indeed, such lipocations do not make pores in the membrane, which depolarise the cell plasma membrane and enable the release of the cytosolic constituents. The chemical formulas of the most well-known penetrating cations of this group are shown in Figure 1. These lipocations, or their derivatives (e.g., alkylated forms, dimers, etc.) are being widely used to deliver antioxidants and other types of chemical moieties to the mitochondria of human cells to improve their physiology. Nevertheless, even the molecules of this type of lipocations could be toxic for cells. In our review, we focus on the side effects of this type of electrophilic carrier.

### 1.1. Triphenyl Phosphonium

Tetraphenylphosphonium and the alkylated derivatives of triphenylphosphonium are probably the most well-studied artificial penetrating cations. Their toxic effects were studied in a variety of experimental systems. When alkylated derivatives of TPP are added to mitochondria, they stimulate respiration. This effect is due to the temporary depolarization of mitochondria due to the passage of a charged molecule through the phospholipid bilayer. Moreover, alkyl-TPP can stimulate the protonophore activity of free fatty acids [8]. At the same time, high concentrations inhibit respiration. That could be due to the direct inhibition of respiratory enzymes or mediation by their detergent action on the mitochondrial inner membrane (see review [9]).At the level of whole cells, we have shown that one of the TPP derivatives, C12TPP, simultaneously induces the expression of multidrug resistance pump (MDR) proteins and also inhibits their activity. The inhibition is not toxic for yeast cells, but the inhibition might be used to enhance the effect of antimycotic drugs which are MDR substrates [10,11]. TPP has been shown to cause hepatotoxicity, but the cellular mechanisms are still not clear. Probably, the liver damage is due to apoptotic cell death because it causes apoptosis in the cultured hepatocyte AML12 cell line [12]. Additionally, one can hypothesise that the apoptosis of liver cells induced by TPP and similar large-headed lipocations is due to the disturbance of cell signalling. It has been shown that several gangliosides with large polar heads and regular lipid tails can induce apoptosis in T-lymphocytes [13,14]. Gangliosides are also known for their ability to alter the ensemble properties of small, ordered lipid–protein domains of plasma membranes [15]; such domains are also called rafts [16]. Similar effects have recently been observed for lysolipids, also with relatively large polar heads and small lipid tails [17]. This result points out that the modification of the raft ensemble properties is not specific to the chemical structure of the particular lipid, e.g., gangliosides, lysolipids. Therefore, TPP might act in a similar manner. Moreover, various cell receptors, and especially death receptors, are raft-associated proteins [18]. Thus, by modifying raft properties, large-headed lipids are able to induce apoptosis even in the absence of specific ligands of death receptors. Thus, the induction of apoptosis and, more generally, the influence on the raft ensemble leading to disturbance of normal cell signalling [19] are, possibly, two more side-effects of penetrating cations. Not surprisingly, when the excess of C12TPP is administered to mice, the reason for its toxic effect is also due to liver damage. It has been shown that it triggers massive cell death in liver zone 3. In fact, this is also not surprising, because such damage is typically induced by phospho-organic compounds [20]. The latter points out that, most likely, the toxicity is induced by the degradation products of C12TPP. It has also been shown that decyl-triphenylphosphonium can be used to selectively kill multiple myeloma cancer cells. Treatment with C10TPP increased intracellular steady-state pro-oxidant levels in stem-like and mature multiple myeloma cells. Ectopic expression of manganese superoxide dismutase is able to suppress the increases in oxidant production induced by C10TPP treatment [21], which also points at the prooxidant activity of C10TPP.Possibly, the anticancer effect of the TPP moiety contributes to the anticancer effect of TPP-linked compounds. Indeed, it is well known that TPP is used to deliver a number of drugs to the mitochondria of tumour cells in order to kill them or inhibit their proliferation. Interestingly, TPP-based mitochondria-targeted anticancer drugs mainly target tumour cells with high membrane potential. Possibly, the latter is due to an increased accumulation of the positively charged compounds in hyper-polarized mitochondria [22].

### 1.2. Dequalinium Chloride (DQ)

Dequalinium is a quaternary ammonium compound with well-documented antibacterial activity. It is being used to treat several types of infections. While being a penetrating cation, it is also capable of lysing bacterial outer membranes [23]. Its targets within the bacterial cells include inhibition of glycolysis, inhibition of F1-ATPase, and protein biosynthesis via interfering with ribosomal activity [23,24]. Similar to the alkylated forms of triphenylphosphonium, DQ does display selective toxicity for cancer cells. The anticancer activity of DQ is due to its ability to target mitochondria, inhibit certain kinases, and affect Ca^2+^-activated K^+^ channels. It specifically targets the mitochondrial membrane of epithelial carcinoma cells, leading to the disruption of cellular energy production. This disruption leads to oxidative stress by reducing glutathione levels and increasing reactive oxygen species levels in a cell-specific manner [25]. DQ also interferes with signalling pathways, such as by downregulating the Raf/MEK/ERK1/2 and PI3K/Akt pathways in NB4 leukaemia cells [26,27,28]. The mitochondrial damage induced by DQ is an early event in its mechanism of action [26]. This damage includes the depletion of mitochondrial DNA [29]. DQ also causes a decrease in mitochondrial membrane potential; it also triggers mitochondrial ROS generation and the depletion of ATP [30].

### 1.3. Pyrvinium and Cyanine

Pyrvinium, a penetrating cation related to cyanine dye, has been used for many decades as an anthelmintic. Similar to other clinically used penetrating cations, its mitochondrial localization and targeting are well documented [31]. In particular, it has been shown that pyrvinium inhibits mitochondrial respiration in parasitic worms [32]. Its mitochondrial accumulation is also responsible for its activity against pathogenic fungi such as Candida auris [33] and Aspergillus fumigatus [34].

Over the past two decades, increasing evidence has emerged showing pyrvinium to be a strong selective anti-cancer molecule in various human cancers in vitro and in vivo [31]. Similar to its anti-parasitic activity, the anti-cancer action of pyrvinium relies on the inhibition of mitochondrial function. There is strong evidence that the inhibition of respiratory complex I is largely responsible for the anticancer activity. Indeed, it was reported that the inhibitory concentrations are in the sub-micromolar range [35,36]. At the same time, it has been noticed that cancer cell lines with depleted mitochondrial DNA are resistant to pyrvinium [35,37]. Complex I is not the only mitochondrial target of pyrvinium. Fumarate reductase [38] and mitochondrial DNA [39] were reported as others. The latter is due to the binding of pyrvinium to G-quadruplex structures, which are more frequent in the mitochondrial DNA than in the nuclear one [40,41]. Mitochondria are not the only targets of pyrvinium. In the cytosol, it binds and activates Casein kinase 1α, leading to the degradation of beta-catenin, the key effector of the WNT signalling pathway. This also contributes to the anticancer activity of pyrvinium [42,43,44]. It also binds and inhibits a number of other non-mitochondrial proteins, including ELAVL1/HuR, a post-transcriptional regulator of a set of genes driving the survival of cancer cells [45,46,47] and the androgen receptor. The latter property has been explored to treat prostate cancer [48,49,50].

Cyanine fluorophores have a high extinction coefficient and relatively long absorption and emission wavelengths. Combined with their low toxicity in biological samples, cyanine fluorophores are promising candidates for biomedical applications [51]. Cyanine fluorophores are used for photodynamic therapy and photothermal therapy, which, due to the high precision of localization of the effect, are very promising methods for treating cancer [52].

### 1.4. Rhodamine

Rhodamine is a fluorescent dye commonly used in laboratory practice to visualise mitochondria. Despite its affinity for mitochondria, its toxicity is attributed to the inhibition of cytosolic antioxidant enzymes, superoxide dismutase (SOD), catalase (CAT), and guaiacol peroxidase (GPOD). It is believed that the inhibition is caused by the degradation products of rhodamine, with degradation being caused by cytochrome P450 [53,54]. Similar to the aforementioned penetrating lipocations, Rhodamine is specifically toxic for carcinoma cancer cells [55]. It has been suggested that the reason for the selective toxicity is due to the fact that these cancer cells have a higher electric potential at their plasma membranes than the normal ones, leading to an increased accumulation of the compound within the cells [56]. However, it should be mentioned that not all cancer types have an increase in plasma membrane (PM) transmembrane potential. Nevertheless, rhodamine was accumulating inside the mitochondria of the carcinoma cells, and its re-localisation to the cytosol was observed shortly before the cell death [55]. This observation suggests that mitochondrial damage is the primary reason for the toxicity of rhodamine.

In addition, rhodamine-based fluorescent labels were shown to induce lipid oxidation that leads, in particular, to an alteration of the dynamics of membrane rafts [57], potentially yielding perturbation of cell signalling, although the mechanical properties of membranes remained almost unaltered [58,59].

Interestingly, it has been reported that certain rhodamine conjugates do possess surfactant activity [60]. Thismeans that, possibly, the penetration of such large molecules through the membrane is accompanied by the disturbance of the integrity of the phospholipid bilayer. Thus, according to our definition of the penetrating cation (see above), it is likely that these conjugates are not true penetrating cations. The abovemay also explain the mitochondrial damage induced by various rhodamines.

### 1.5. Berberine, Palmatine and Sanguinarine

Berberine, palmatine and sanguinarine are chemically related compounds produced by plants as a defence against various parasites. Not surprisingly, they have several intracellular targets and affect cellular physiology by a number of independent means. First, berberine and sanguinarine bind DNA and inhibit DNA synthesis [61]. Palmatine was also shown to specifically induce DNA strand breakage in cultured cancer cells [62]. Isoquinoline alkaloids can interact with DNA in a variety of ways: by binding to phosphate groups, or to a small groove, or as intercalators [63]. Possibly, that explains why berberine, palmatine and sanguinarine target DNA. Berberine, by binding to histone-DNA complexes, can interfere with vital cellular processes such as cell division and activate apoptosis in living cells, leading to the selective death of cancer cells [64,65].

Apart from binding DNA, these compounds interact with and inhibit several extra- and intracellular proteins. Acetylcholine esterase, butyrylcholinesterase, and choline acetyltransferase are inhibited by them, as are the receptors alpha 1- and alpha 2-adrenergic, nicotinergic, muscarinergic, and serotonin2 receptors [61].

Sanguinarine [13-methyl(1,3)benzodioxolo(5,6-c)-1,3-dioxolo(4,5)phenanthridinium] is more toxic than berberine or palmatine. The additional toxicity of sanguinarine is mostly due to its inhibition of the Na^+^-K^+^-ATPase transmembrane protein [63]. Sanguinarine has also been shown to cause cell membrane damage by lipid peroxidation by free radicals, including ROS and reactive nitrogen species (RNS). It also possesses DNA polymerase inhibition activity and causes the accumulation of pyruvate due to increased glycogenolysis [66].

### 1.6. E-4-(1H-indol-3-ylvinyl)-N-methylpyridinium Iodide (F16)

A novel penetrating cation, F16 has been recently shown to possess uncoupling activity. It was also reported that it specifically accumulates in the mitochondria of carcinoma cells and inhibits their growth. It has been concluded that mitochondrial toxicity by the means of uncoupling mediates the anticancer activity of F16 [67]. F16 conjugates with ursolic, oleanolic, maslinic, and corosolic acids, which show increased toxicity to cancer cells. This toxicity is also due to an increase in ROS that occurs during the action of the conjugates on mitochondria [68]. Work is underway to study the anticancer toxicity of F16 derivatives; for example, it has been shown that 5-Br-7MeF16 (5BMF) has a low IC50 of ∼50 nM (to H2228 cells) and a high cancer to normal cell selectivity index of 225 [69].

### 1.7. Methylene Blue

Methylene blue (MB) induces non-toxic hydrogen peroxide production and, in this way, protects mitochondria, in particular, from rotenone toxicity. For this reason, methylene blue displays a broad range of neuroprotective activity. Its therapeutic effect is also due to the activation of mitochondrial biogenesis, the latter being mediated by the Nrf2/ARE signalling cascade. It is believed that the cascade is triggered by H_2_O_2_ generation induced by methylene blue [70,71]. Interestingly, similar to the other penetrating cations, methylene blue is selectively toxic to cancer cells. It was shown that lung cancer cells treated with methylene blue show reduced activity of Hsp70, the protein that is essential for their survival [72]. It is not clear whether this is a direct inhibition or a consequence of mitochondrial damage or H_2_O_2_ production. MB is able to disrupt NO production by inhibiting guanylyl cyclase and endothelial NO synthase [73]. MB can accumulate in the mitochondria and lysosomes of HaCaT cells and demonstrate phototoxicity [74].

### 1.8. Sepantronium Bromide (Ym155)

In preclinical studies, the imidazolium-based compound Ym155 displayed selective suppression of the growth of many types of cancer cell lines. It was discovered as a result of screening for inhibitors of survivin expression. However, its biomolecular target remains unknown [75]. The reason for that has started to become clear only recently. It has been shown that, being a penetrating cation, it accumulates in mitochondria and binds to mitochondrial DNA, causing energy depletion in the cells [76]. Ym155 generates ROS in mitochondria, causing DNA damage and inhibiting survivin. This mediates the cytotoxic effects of Ym155 on triple-negative breast cancer TNBC cells through both apoptotic and necrotic pathways [77].

#### 1.8.1. Metformin and Doxorubicin

The compounds metformin and doxycycline differ in their way of penetration into the cells from other chemicals discussed in the review. Nevertheless, their therapeutic effects are similar to those of the other penetrating cations.

#### 1.8.2. Metformin

Metformin, a derivative of guanidine, has been used for decades in the treatment of type 2 diabetes. Metformin has a pKa of 12.4 and thus, under physiological conditions, it exists in a protonated form. It has been reported that metformin enters cells via organic cation transporters (Octs) [78] and then targets mitochondria. For this reason, mitochondria play a key role in both pharmacological and toxic actions of metformin [79]. A number of studies have shown that metformin selectively inhibits the growth of several types of cancer cells [80]. It has been suggested that the primary reason for the anticancer activity of metformin is inhibition of mitochondrial energy production [80]. The latter is consistent with the observation that metformin inhibits complex I in the mitochondrial respiratory chain [79]. Metformin has also been shown to induce ROS formation, thus stimulating redox signalling mechanisms [80]. It has been suggested that this is due to complex I inhibition [81]. Formation of ROS may be a negative side-effect of this cation if it occurs in normal cells; it was shown that agents that depolarize the mitochondrial membrane and prevent ROS formation in cells under oxidative stress-associated conditions exhibit neuroprotective activity [82,83]. There is also evidence that metformin directly activates AMP-dependent protein kinase (AMPK), the key sensor of cellular energy status, via the lysosomal AXIN-dependent pathway [84], leading to the suppression of cell growth. It has also been reported that metformin inhibits PI3K/AKT signalling, the pathway which is essential for cell growth and proliferation [80]. Interestingly, it has been shown that metformin induces apoptosis in ovarian cancer cells by changing the balance of Bax and Bcl-2 proteins in an AMPK-independent manner [85]. Another target of metformin is DNA methylation. It has been reported that the addition of metformin leads to a decrease in S-adenosylmethionine levels in *C. elegans*, which forces the H3K4 methyltransferase/demethylase complex to downregulate the targets [86]. To summarise, while mitochondria are the primary target of metformin, the compound also affects a set of non-mitochondrial targets within a cell.

#### 1.8.3. Doxorubicin (Dox)

Doxorubicin (Dox) is an antibiotic, which is produced by Streptomyces peucetius bacterium. pKa of Dox equals 8.01, so most Dox molecules are charged at physiological pH [87]. To our knowledge, it has not been shown directly that Dox in protonated form is capable of crossing the lipid bilayers. Still, it has been shown that, when added to MDA-MB-435 cancer cells, it co-localizes with mitochondria [88]. Its ability to selectively damage mitochondria (see below) also points out that protonated Dox is a penetrating cation.

Dox is commonly used to treat different cancers. It has been reported that Dox-induced killing of cancer cells is mediated by intercalation within DNA base pairs, preventing the synthesis of both DNA and RNA and inhibiting topoisomerase II. Dox also intercalates mitochondrial DNA [89]. This can cause breaks in the mitochondrial DNA, either in one strand or both. Moreover, doxorubicin combined with iron also causes damage to DNA through the production of harmful ROS [90,91,92].

There was an attempt to increase the mitochondrial accumulation of Dox. For this purpose, the molecule of Dox was linked to TPP. The attempt was obviously successful. It was demonstrated that, unlike Dox, chimeric Dox-TPP accumulated not only in the mitochondria of cancerous MDA-MB-435 cells, but also in mitochondria of MDA-MB-435 wild-type cells, the ones with lower mitochondrial membrane potential [88]. Later, it was shown that Dox-TPP-containing nanoparticles of various types have a great potential to selectively eliminate cancer cells by targeting their mitochondria [93,94,95].

Treatment with Dox can increase the risk of death for cancer patients because of its cardiotoxicity. Cardiotoxicity can lead to the development of cardiomyopathy and, ultimately, congestive heart failure. Again, one of the mechanisms responsible for Dox-induced cardiotoxicity is impaired mitochondrial function and ROS generation in heart cells. It has been demonstrated that mitochondria are one of the major sites for Dox-induced oxidative stress and cellular damage [96].

Dox-induced ROS generation activates four proteolytic systems: (1) the ubiquitin proteasome system (UPS), (2) calpain, (3) caspase-3, and (4) autophagy. (1) Polyubiquitinated damaged proteins are degraded by UPS. It has been shown that treatment with Dox can increase this protein breakdown in cardiac muscle, which can ultimately lead to damage in the heart [97]. (2) Altered levels of Ca^2+^ are also one of the mechanisms involved in Dox-induced cardiotoxicity. Dox treatment causes calcium overload, which increases the activity of a protein calpain. This protein cleaves cytoplasmic and nuclear substrates, leading to apoptosis [98]. (3) Caspases are enzymes that play a role in programmed cell death. Studies have shown that treatment with Dox can activate the apoptosis regulators, resulting in the activation of caspase-3 in cardiac muscle, leading to cell death [99]. Consistent with this, inhibiting caspase-3 activity can help to reduce the cardiotoxiceffects of Dox [100]. (4) The level of autophagy is low in the heart muscle but, under stress, autophagy is increased as a response [101]. Dox treatment increases the Beclin-1/Bcl-2 ratio, which is responsible for activation of both autophagy and apoptosis [89].

## 2. Discussion

Table 1 presents the summary of known effects of the penetrating cations. The table shows that their only common features are the disruption of phospholipid bilayers and the selective toxicity towards cancer cells.

The feature not shown in the table is that all of the compounds inflict damage on mitochondria. Although it is apparent that small lipophilic cations are mitochondrial toxins, the exact basis for their toxicity is difficult to pinpoint. It has been noticed that the relatively hydrophobic compounds (Rh123) affect electron transport and ATP synthesis, while the relatively hydrophilic ones perturb the matrix proteins and also inflict mito-DNA damage ([102,103,104,105,106] and Table 1). One can speculate that the membranophilic nature of the cations will also affect the phospholipids of the mitochondrial inner membrane. It is well known that typical lipids of biological membranes have conical (small polar head and large hydrophobic tails) or cylindrical (approximately equal cross-sections of polar head and hydrophobic tails) molecular shapes. At the same time, they have negative or zero spontaneous curvature [107,108]. It has been reported that such molecular shapes prevent the spontaneous formation of pores in the planar membranes [5,109]. On the contrary, membranes containing molecules having an inverse conical shape (large polar head, small hydrophobic tail) are prone to forming pores [110]. The polar groups of lipophilic cations shown in Figure 1 are likely to have an affinity for the polar heads of the lipid bilayer. Indeed, hydrophobic parts of the cations tend to avoid contact with water and accumulate inside the hydrophobic membrane core with their aromatic groups being mostly distributed to the polar/hydrophobic interface of a lipid monolayer. Each of the cations shown in Figure 1 has relatively small hydrophobic parts and large polar or/and aromatic parts. Upon incorporation into the membrane, they, obviously, disturb the ordering of the phospholipid molecules, and this disturbance might cause an increase in the permeability of various small molecules through the membrane either via packing defects or by pore formation. In fact, non-penetrating cations, such as benzalkonium, disturb the lipid packing in a cholesterol-sensitive manner [111] and inflict the same type of damage on the lipid bilayers as the penetrating cations. This property makes them powerful antibacterial agents. Indeed, while the outer membrane of eukaryotic cells is protected by a high content of sterols, bacterial membranes typically lack sterols and thus are vulnerable to the disruption of ordering of the phospholipid molecules. For example, imidazolium-based ionic liquids can be used as antibacterial agents. Our preliminary data indicate that they do not penetrate through the bilayer, and thus their antibacterial effect is mostly due to their membrane-disrupting action.

Another common feature of the discussed penetrating cations is their anticancer activity, especially against the carcinoma cells. This was noticed a couple of decades ago [25], and further studies confirmed this observation (see above). The reason for that might be due to the fact that typically, for energy supply, cancer cells rely on glycolysis (Warburg effect). This type of metabolism is typically accompanied by the inhibition of mitochondrial respiration. As a certain level of mitochondrial membrane potential is required for cell survival, such cells use mitochondrial ATP synthase in a reverse fashion for the potential generation. As this type of the potential generation is much less powerful than respiration, even a relatively minor increase in the proton conductivity of the mitochondrial inner membrane might be lethal for the cell. Such an increase might be induced by the accumulation of the lipophilic cations in the mitochondrial inner membrane. Indeed, yeast cells that lack functional mitochondrial DNA and rely solely on glycolysis are much more sensitive to conventional protonophores than those with intact mito-DNA [112]. The same is true for cancer cells: at least certain cancer cell lines are more sensitive to uncouplers than normal cells [113]. As all of the penetrating cations in one way or another decrease the membrane potential, this may explain their anti-cancer activity.

An alternative explanation of the anti-cancer activity of the penetrating cations is that many types of cancer cells display an elevated level of the membrane potential. Mitochondria of such cells possess fully functional respiratory chains and, at the same time, non-functional ATP synthase. The inhibition is due to the expression of a special inhibitory protein, IF1. Apparently, the increased charge of mitochondria presumes an increased level of penetrating cation accumulation. As some of them inhibit specific mitochondrial enzymes or bind mitochondrial DNA, such accumulation might render these types of cancer cells more sensitive to the cations than the normal ones.

Another possible reason for the sensitivity of cancer cells to the penetrating cations is that many types of cancer cells show elevated activity of multidrug resistance pumps (MDRs). The reason is that these pumps extrude various chemicals used for chemotherapy. We have recently shown that a set of penetrating cations, alkylrhodamines, are inhibitors of MDRs. In the case of yeast, they display strong synergy with antimycotic drugs [114]. Possibly, in the case of cancer cells the penetrating cations act in the same way.

## 3. Conclusions

To summarise, the analysis of the literature points out that the only common features of all the aforementioned penetrating cations are their anticancer activity and damage to mitochondria. While each of the cations damages mitochondria in its own way, they all have something in common in this respect, namely the disruption of the ordering of the phospholipid molecules of the inner mitochondrial membrane. This, most likely, contributes to their selective toxicity toward cancer cells. Indeed, it is well known that the depletion of energy (ATP) in cancer cells is a commonly used strategy to treat tumours (reviewed in [115,116]). While the reason for the increased sensitivity of the cancer cells to penetrating cations is not exactly clear, the very fact, in our opinion, is beyond doubt.

## Figures and Tables

**Table 1:** Toxic effects of the most well-studied penetrating cations. See text for details.

	Triphenyl phosphonium	Dequalinium chloride	Pyrvinium	Rhodamine	Berberine	F16	Methylene blue	YM155	Doxorubicin	Metformin
Proton conductivity	√					√				
Inhibition of respiratory Chain	√		√	√			√			√
DNA damage		√			√			√	√	
Inhibition of MDR pumps	√									
Inhibition of soluble (non-membrane bound) proteins	√	√	√	√	√		√	√	√	√
Disruption of phospholipid bilayers	√	√	√	√	√	√	√	√	√	√

## Data Availability

Not applicable.
